# Persistence of Pathological Distribution of NK Cells in HIV-Infected Patients with Prolonged Use of HAART and a Sustained Immune Response

**DOI:** 10.1371/journal.pone.0121019

**Published:** 2015-03-26

**Authors:** Mario Frias, Antonio Rivero-Juarez, Ana Gordon, Angela Camacho, Sara Cantisan, Francisca Cuenca-Lopez, Julian Torre-Cisneros, Jose Peña, Antonio Rivero

**Affiliations:** 1 Infectious Diseases Unit, Hospital Universitario Reina Sofia de Córdoba, Instituto Maimonides de Investigación Biomédica de Córdoba (IMIBIC), Cordoba, Spain; 2 Immunology Unit, Hospital Universitario Reina Sofia de Córdoba, Instituto Maimonides de Investigación Biomédica de Córdoba (IMIBIC), Cordoba, Spain; Karolinska Institutet, SWEDEN

## Abstract

**Objective:**

A prospective analysis of the distribution of NK subsets and natural cytotoxicity receptors (NKp30/NKp46) in HIV patients with long-term HAART use and sustained virological and immunological response.

**Methods:**

The main inclusion criteria were: at least 3 years’ receipt of HAART; current CD4^+^ count ≥ 500 cells/mm^3^; undetectable viral load for at least 24 months; no hepatotropic virus co-infection. Percentages of CD56^dim^, CD56^bright^ NK cells and CD56^neg^ CD16^+^ cells were obtained. Expression of the NCRs, NKp30 and NKp46 was analysed in CD56^+^ cells. Thirty-nine infected patients and sixteen healthy donors were included in the study.

**Results:**

The percentages of total CD56^+^ and CD56^dim^ NK cells were significantly lower in HIV-infected patients than in healthy donors (70.4 vs. 50.3 and 80.9 vs. 66.1 respectively). The percentage of total CD56^+^ NK cells expressing NCR receptors was lower in HIV patients than in healthy donors (NKp30: 25.20 vs. 58.63; NKp46: 24.8 vs. 50.59). This was also observed for CD56^dim^ and CD56^bright^ NK cells. Length of time with undetectable HIV viral load was identified as an independent factor associated with higher expression of NKp30 and NKp46.

**Conclusion:**

Despite the prolonged and effective use of HAART, HIV-infected patients do not fully reconstitute the distribution of NK cells. Length of time with an undetectable viral load was related to greater recovery of NKp30/NKp46 receptors.

## Introduction

Replication of the human immunodeficiency virus (HIV) triggers an abnormal pathological redistribution of the natural killer (NK) cells [1–2], which reduces the percentage of the cytolyticCD56^dim^ subpopulation and increases that of the dysfunctional CD56^neg^NK population [1, 3–7]. Furthermore, HIV replication leads to a reduced expression of the natural cytotoxicity receptors (NCR), NKp30, NKp44 and NKp46 [1, 8–9]. The reduction in the number of CD56^+^NK cells and down regulation of the activating receptors may limit the functionality of the innate immune response, so compromising the response to possible opportunistic infections or tumours [2,8].The suppression of HIV replication therefore could, in theory, imply a reversal of such abnormalities in the NK cells.

In the context of the suppression of viral load through highly active antiretroviral therapy (HAART), several authors have studied the influence of changes that may occur on NK cells, with discrepant results. On the one hand, the recovery of NK cell subsets and activating receptors to levels similar to those of healthy individuals has been reported when HIV patients have been on HAART for two years [9]. Other studies however have found incomplete NK subset recovery after initiating HAART when compared with T-cell recovery during the early months of therapy [10].

Given this controversy, further studies are needed to clarify what occurs in NK cells, which is an important arm of innate immune response in HIV infection. Furthermore, there are no studies of the frequency, phenotype and extent of recovery of NK cells in the context of prolonged and effective use of HAART.

## Material and Methods

### Study design

We designed a prospective study in order to analyse the distribution of NK cell subsets and NCRs in HIV patients with prolonged use of HAART and a sustained virological/immunological response.

### Patients and variables collected

Chronic HIV-infected patients in follow-up in the Infectious Diseases Unit of the Hospital Universitario Reina Sofia (Cordoba, Spain) between December 2013 and April 2014were included. The main inclusion criteria were in receipt of HAART for at least 3 years; current CD4^+^count ≥ 500 cells/mm^3^,confirmed on two consecutive determinations; an undetectable viral load for at least 24 months, measured by PCR (CobasTaqMan, Roche Diagnostic Systems Inc., Pleasanton, CA, USA), with detection limit set at 20 IU/mL; and no hepatotropic virus co-infection. Data relating to age, sex, current CD4 count, nadir CD4 count, increased CD4, CD8, CD4/CD8 ratios, length of time with HAART and length of time with undetectable viral load were also collected. Healthy donors were included as controls and their data was collected and analyzed in parallel, under the same conditions and experiments as the HIV-infected patients.

### NK subsets and NCR evaluation

Peripheral blood mononuclear cells (PBMCs) were isolated in 3 mL EDTA tubes by density gradient centrifugation (Ficoll-Hypaque). Isolated PBMCs were cryopreserved in liquid nitrogen, using a freezing medium composed of Fetal Bovine Serum (FBS) and 10% DMSO until analysis. NK cells and their subpopulations were defined on the basisoflive cells (Propidium Iodide) and the expression of CD3, CD16 and CD56 receptors in the peripheral blood lymphocyte region. Percentages of CD56^dim^ (CD3^neg^, CD56^pos+^, CD16^pos/neg^), CD56^bright^(CD3^neg^, CD56^pos+++^, CD16^pos/neg^) and CD56^neg^CD16^pos^(CD3^neg^, CD56^neg^, CD16^pos^) cells were obtained. The CD56^pos^-CD16^pos/neg^ cells were analysed for the expression of the natural cytotoxicity receptors, NKp30 and NKp46 (S1 Fig.).PBMCs were stained using anti-CD3-Vioblue (BW264/56), anti-CD16-APCVio770 (VEP13), anti-CD56-PEVio770 (AF12-7H3), anti-NKp30 (AF29-4d12), and anti-NKp46-APC (9E2). Propidiumiodide (PI) solution was used to assess cell viability. All antibodies and the PI were obtained from MiltenyiiBiotec (Germany). For acquisition, the MACSQuant (MiltenyiiBiotec, Germany) system was used. Data were analyzed using FlowJo Software (Tree Star, OR, USA).

### Statistical analysis

Categorical variables were expressed as numbers of cases (percentages), and continuous variables as medians (interquartile range). Receptor expression and subpopulations were represented as percentages (median with IQR). Furthermore, normalized mean fluorescence intensity (NMFI) values were calculated for NKp30/46 receptors. Continuous variables were analysed using the Mann Whitney U-test; categorical variables were analysed by applying Fisher’s exact test. The Spearman correlation test was used for bivariate analysis. Five linear regression models were performed to identify independent predictors of the frequencies of NK cell subsets (total CD56^+^, CD56^dim^, CD56^bright^ NK cells) and natural cytotoxicity receptor expression (NKp30 and NKp46). The coefficients (b) of the models were shown as adjusted coefficients. The analysis was performed using the SPSS statistical software package, version 18.0 (IBM Corporation, Somers, New York, USA).

Among HIV-infected patients, the relationship between percentages of NK cells (subsets, NKp30^+^ and NKp46^+^ cells) was studied, as well as various clinical variables, such as AIDS/non-AIDS-defining conditions, nadir CD4^+^ count, current CD4^+^ count, increase in CD4^+^, age, and length of time with undetectable HIV viral load. For each variable, the patients were sorted into two groups with respect to the median of the variable.

### Ethical statement

The study was designed and performed according to the Helsinki Declaration and was approved by the ethics committee of the Reina Sofía University Hospital, Cordoba, Spain. All of the patients provided written informed consent before participating in the study and gave permission for biological samples to be stored and processed.

## Results

### Study population

Thirty-nine infected patients and sixteen healthy donors were included in the study. The median age of patients was 43 years (IQR, 35–51 years), and 31 (79.5%) were men. Twelve (30.7%) patients had AIDS-defining conditions in the past. 38 (97.4%) had risky sexual practices and 1 of them had had parenteral drugs in the past. The nadir CD4^+^ count (median) was 258 cells/mL (IQR, 100–342 cells/mL). CurrentCD4^+^ and CD8^+^ counts were 652 cells/mL (IQR, 594–880cells/mL) and 741 cells/mL (IQR, 586–1034 cells/mL), respectively. Length of time on HAART was 93 months (IQR, 53–139 months), and length of time with undetectable HIV viral load was 85 months (IQR, 47–128 months). The median age of healthy donors was 36.50 years (IQR, 29.75–47.25 years), and 10 (62.5%) were men.

### NK cell distribution and NCR expression in HIV-infected patients as compared with healthy donors

**Fig 1:**
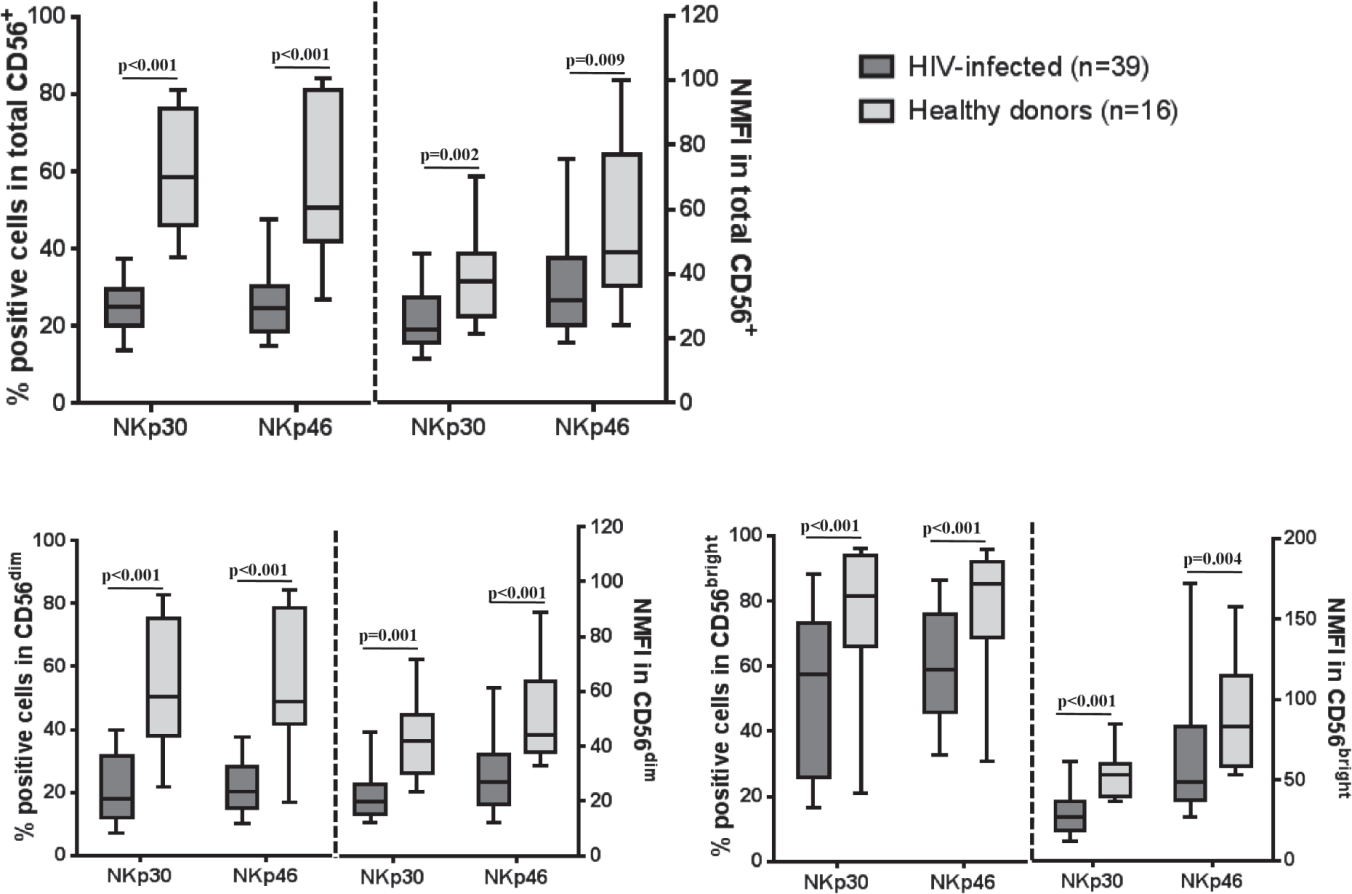
Flow cytometric analysis of NKp30 and NKp46 natural cytotoxicity receptors. The figure shows the percentage of cells expressing NKp30 and NKp46 and normalized mean fluorescence intensity values for total CD56+ NK cells and CD56^dim^and CD56^bright^ subsets. The values are expressed as medians with interquartile range. The analysis compared HIV-infected patients and healthy donors.

Total CD56^+^ NK cell counts (percentage among CD3^neg^ cells) were lower in HIV-infected patients than in healthy donors (50.3 [37.6–65.2] vs. 70.4 [48.3–81.1]; p<0.001). CD56^dim^ percentages were significantly lower in HIV-infected patients than in healthy donors (66.1 [52.9–75.8] vs. 80.9 [75.6–82.9]; p<0.001) and the percentage of CD56^neg^CD16^+^cells was higher in HIV-infected patients than in the control group (28.9 [18.5–36.9] vs. 13.7 [10.7–16.7]; p<0.001). No differences in percentages of CD56^bright^ NK cells were found between the HIV-infected group and healthy donors (5.88 [3.5–8] vs. 5.18 [4.5–7.2]; p = 0.902). The percentage of CD56^+^ NK cells expressing NKp30 and NKp46 receptors was lower in HIV patients than in healthy donors (Fig. 1). Furthermore, when the normalized mean fluorescence intensity values of the two groups were compared, HIV patients had a lower density of NKp30/NKp46 than healthy donors. (Fig. 1). The same phenomenon was also found for CD56^dim^ andCD56^bright^cells. (Fig. 1).

### Factors associated with higher NCR expression in HIV-infected patients

There was no correlation between the percentage of NCR expression and nadir CD4^+^count, current CD4^+^ count, increased CD4^+^, AIDS/non-AIDS defining conditions or age. However, a higher and statistically significant percentage of NK cells expressing NKp30 and NKp46 (Table 1) was found in patients who had achieved more than 85 months of undetectable viral load. There was also no correlation between these variables and frequencies of NK cell subsets (S1 Table).

**Table 1:** Percentages of NKp30+ and NKp46+ cells among HIV-patients according to various clinical variables.

**Clinical variable**		**NKp30+ cells**		**NKp46+ cells**	
Age (years)	<43	23 (19.4–27.9)	p = 0.234	21.8 (17.9–30.7)	p = 0.379
	≥43	27.1 (23–30.6)		25.9 (20.7–29.9)	
AIDS in past (criteria)	AIDS	28.4 (22.9–30.7)	p = 0.233	24.9 (19.7–30.1)	p = 0.730
	Non-AIDS	23.6 (19.4–27.5)		24.8 (18.1–30.5)	
Nadir CD4 (cel/mL)	<258	27 (22.9–31.2)	p = 0.246	24.9 (19.8–30.4)	p = 0.644
	≥258	23.1 (19.1–29.1)		24.5 (17.5–31.5)	
Current CD4 (cel/mL)	<652	23.4 (19.3–29.4)	p = 0.428	22.6 (18.2–30.2)	p = 0.607
	≥652	26.5 (21.3–32)		24.9 (20.2–30.4)	
Increase CD4 (cel/mL)	<458	24.7 (19.3–28.7)	p = 0.627	22.6 (17.5–32.8)	p = 0.513
	≥458	25.6 (21.3–31.2)		25.9 (20.2–29.4)	
UVL^a^ (months)	<85	23 (18.5–27.1)	**p = 0.007**	19.8 (16.3–24.8)	**p = 0.003**
	≥85	27.6 (23.2–33.1)		28.1 (24.6–31.6)	
Healthydonors		58.6 (46–76.2)		50.5 (41.9–80.9)	

Percentages of NCR expression are presented as median and interquartile range (Q1-Q3). The percentage of “NKp30^+^ cells” and “NKp46^+^ cells” was calculated with respect to the total population of CD56^+^NK cells. The p values were obtained by the Mann–Whitney U test.

^a^undetectable viral load

**Fig 2:**
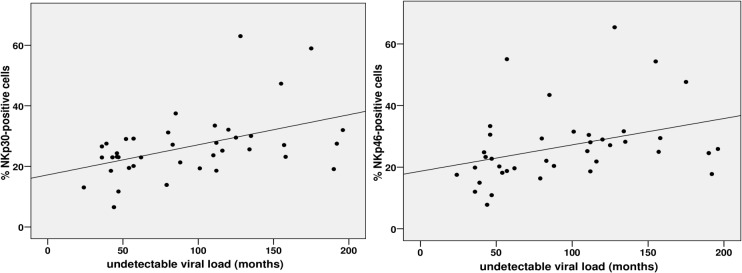
Graph for bivariate analysis between time with undetectable viral load and frequency of NKp30/NKp46+ cells. The percentage of NK cells expressing NKp30 (Spearman rho, r = 0.47 p = 0.002) and NKp46 receptors (Spearman rho, r = 0.45 p = 0.004) correlate positively with length of time with undetectable viral load.

There was a positive correlation between the percentage of NK cells expressing NKp30 (Spearman rho, r = 0.47 p = 0.002) and NKp46 receptors (Spearman rho, r = 0.45 p = 0.004) and length of time with undetectable viral load (Fig. 2). Furthermore, a multivariate linear regression model identified length of time with an undetectable viral load as the only independent factor for NKp30 and NKp46 expression (Table 2).

**Table 2:** Multivariate linear regression model for percentage of NKp30+ and NKp46+ cells.

	NKp30^+^ cells			NKp46^+^ cells		
	B^a^	95% CI	p	B	95% CI	p
Age	−0.186	−0.508; 0.155	0.286	−0.165	−0.570; 0.220	0.373
Nadir CD4^+^	−0.254	−0.039; 0.006	0.148	−0.239	−0.044; 0.010	0.200
Δ CD4+^b^	−0.237	−0.019; 0.004	0.171	−0.231	−0.022; 0.005	0.208
Undetectable VL^c^	0.549	0.044; 0.195	**0.003**	0.434	0.016; 0.196	**0.022**

^a^ Coefficient;

^b^ Increase CD4^+^ = current CD4^+^ count-Nadir CD4^+^ count;

^c^ length of time with an undetectable viral load (months).

Model R^2^ values for NKp30^+^ cells (R^2^ = 0.287) and NKp46^+^ (R^2^ = 0.192)

## Discussion

Our results suggest that, in spite of the prolonged use of HAART, a long period with an undetectable viral load and a remarkable increase in CD4^+^ count, HIV-infected patients were unable to completely restore the innate immunity mediated by NK cells, and showed a decreased proportion of CD56^+^ cells, particularly the cytolytic CD56^dim^ subpopulation, and low expression of NCRs.

In addition, we also found an increase in the percentage of CD56^neg^-CD16^+^ cells in HIV-infected patients, although, in our study, we cannot consider them to be specific NK cells. Indeed, there remain many unanswered questions about the phenotype, function, and biology of CD3^neg^CD56^neg^CD16^+^ cells and the lack of NK cell lineage-specific markers makes it difficult to study this NK cell subset.

In this respect, there are studies that have described this subset as cells with impaired or altered cytolytic function [11]. However, recent studies have shown, based on the expression of CD7, that CD56^neg^-CD16^+^ cells in HIV infection are a mixed population of myeloid and NK cells, where CD7^+^CD56^neg^CD16^+^ are mature NK cells without a significantly altered phenotype [12,13]. Further analysis is required to understand what happens to these cells in the context of HIV infection.

In other studies that studied the recovery of NK cells in the context of HIV, Michaëlsson et al. [14] analyzed the reconstitution of NK cells in patients with and without treatment. Although the follow-up was only one year, they did not find clear differences in the frequencies of NK subsets and percentages of NKp30/46^+^ cells between those patients who had been treated and those who had not.

Chemini et al.[10] analyzed NK cell compartments in relation to CD4 recovery in 21 HIV-infected subjects who were followed to <50 copies/ml after starting antiretroviral therapy (ART) and were observed for 52 weeks of sustained suppression. Although the CD4 count increased in all subjects in response to ART, the restoration of total NK cells was incomplete even after 52 weeks of this therapy. In our study, 20 patients received HAART for over 93 months and HIV RNA suppression was maintained for over 85 months, with a median CD4^+^recovery of 458 cells/mL; nonetheless the distribution of NK cell populations was not restored. These results suggest that the recovery of innate immunity after very prolonged viral suppression (over 7 years) is still incomplete and does not reach healthy levels. Innate immunity is known to play a fundamental role in the early defence against pathogens and to exert regulatory control on adaptive responses downstream. The incomplete recovery of innate immunity may lead to patients remaining susceptible to tumours and various viral infections [15–16].

In our study, the only factor associated with any degree of reconstitution of the NK cells was length of time with an undetectable HIV-1 RNA. We observed a positive correlation between the percentage of NK cells expressing NKp30 and NKp46 receptors and length of time with an undetectable HIV-1 viral load. Indeed, patients with more than 85 months showed higher percentages of NKp30- and NKp46-positive cells than those with less than 85 months. In this regard, a greater number of cells positive for these receptors may imply a better response and NK cell activity [1,17]. This finding suggests that a more prolonged and effective antiretroviral therapy may lead to restoration of innate immunity in the long-term, although the difference between the percentage of NCR^+^ cells in groups of patients with more than 85 months of viral suppression and healthy donors remains unbalanced.

In this work, CD4^+^countand CD4^+^ increase from basal were not related to the extent of NK cell reconstitution, which suggests that simply determiningtheCD4^+^ cell count alone in quantitative terms in order to assess the immune status of HIV-infected patients provides only limited information. Thus, Bisio et al. [18] observed that HIV-infected patients with low CD4^+^ cell counts and AIDS-defining opportunistic infections had a differential expression of NK cell activating receptors when compared with HIV-infected patients with similar nadir CD4+ cell counts but who had never had an AIDS-defining condition.

The present study has several limitations. Firstly, the low number of patients included may not have enough statistical power to detect differences between HIV groups of patients on the basis of various clinical variables, such as AIDS/non-AIDS status. Secondly, other potentially related variables, such as length of time with active HIV infection, were not collected. Lastly, functional assays were not performed.

In conclusion, despite the prolonged and effective use of HAART, HIV-infected patients do not reconstitute the pathological NK cell distribution associated with HIV infection. In our study, length of time with undetectable viral load was associated with the greater recovery of NKp30/NKp46 receptors of natural killer cells. Studies evaluating the impact of prolonged HAART on NK cell restitution are needed.

## Supporting Information

S1 Fig Flow cytometric gating strategy for analysis of NK cell subsets and NCR expression. (A) PBL gating was performed on the basis of FSC and SSC parameters. CD56^+^ cells and CD56^dim^, CD56^bright^ and CD56^neg^ CD16^+^ subpopulations were defined according to their expression of CD3, CD16 and CD56 in the PBL region and propidium iodide (PI) was used to assess cell viability. Unstained samples were used as negative controls for all receptors. (B) Dot-plots represents expression of NKp30 and NKp46 in CD56^+^ cells.
(PPT)

S1 Table Percentages of NK subsets among HIV-infected patients, according to various clinical variables. Percentages of NK subpopulations are presented as median and interquartile range (Q1-Q3). The percentage of “Total CD56^+^” was calculated with respect to CD3^neg^ cells. The p values were obtained by the Mann–Whitney U test. Legend: ^a^ undetectable viral load.
(DOC)
