# Supplementary figures and images for: Persistence of Pathological Distribution of NK Cells in HIV-Infected Patients with Prolonged Use of HAART and a Sustained Immune Response
Source: PLoS One. 2015 Mar 26;10(3):e0121019. doi: 10.1371/journal.pone.0121019 (PMC4374841; doi:10.1371/journal.pone.0121019)

## Slide 1
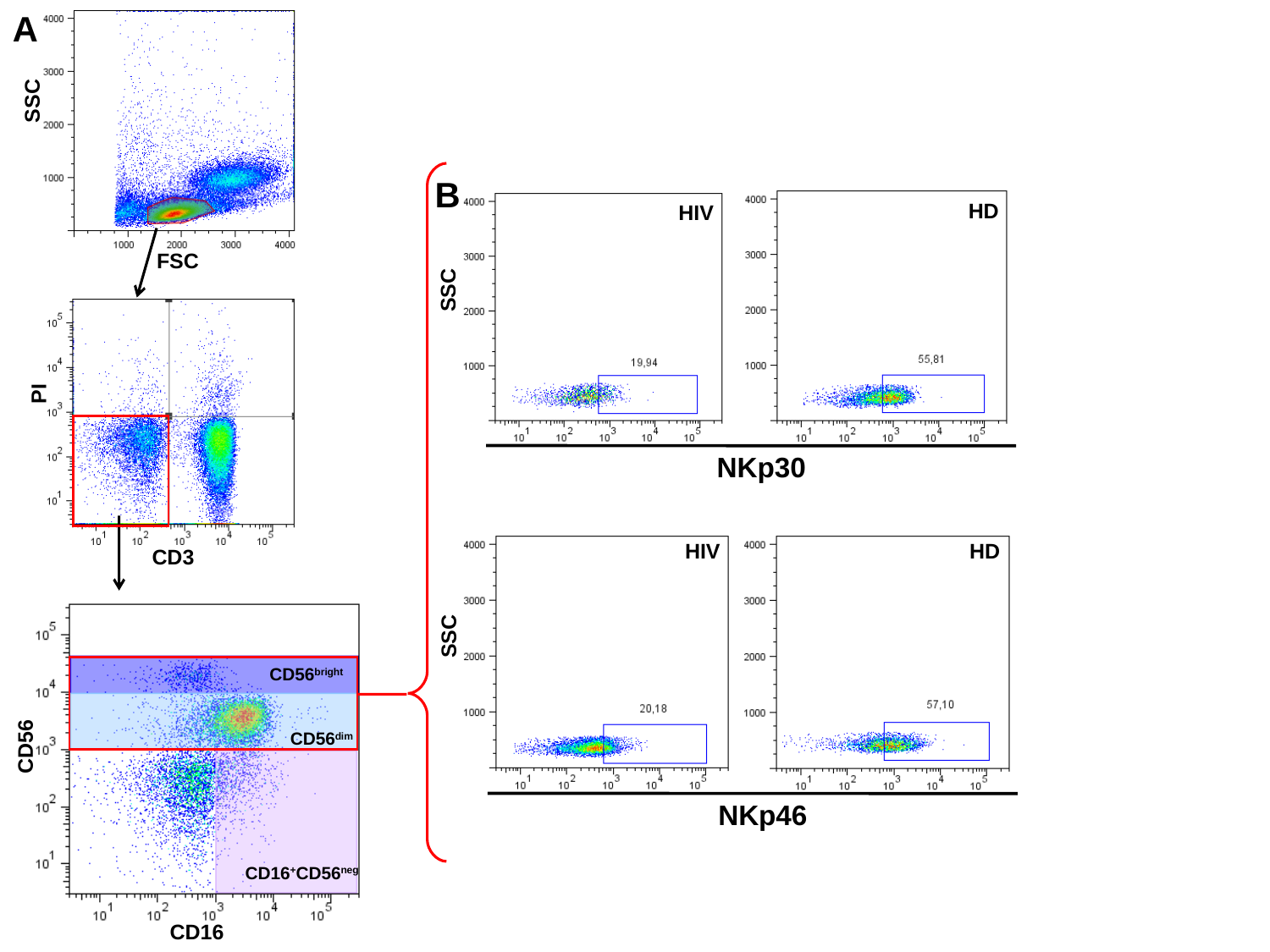

A
SSC
B
HD
HIV
FSC
SSC
PI
NKp30
HD
HIV
CD3
SSC
CD56bright
CD56dim
CD56
NKp46
CD16+CD56neg
CD16

Supplement: S1 Fig — (A) PBL gating was performed on the basis of FSC and SSC parameters. CD56+ cells and CD56dim, CD56bright and CD56neg CD16+ subpopulations were defined according to their expression of CD3, CD16 and CD56 in the PBL region and propidium iodide (PI) was used to assess cell viability. Unstained samples were used as negative controls for all receptors. (B) Dot-plots represents expression of NKp30 and NKp46 in CD56+ cells. (PPT) [file pone.0121019.s001.ppt]
