# Supplementary material for: Persistence of Pathological Distribution of NK Cells in HIV-Infected Patients with Prolonged Use of HAART and a Sustained Immune Response
Source: PLoS One. 2015 Mar 26;10(3):e0121019. doi: 10.1371/journal.pone.0121019 (PMC4374841; doi:10.1371/journal.pone.0121019)
Supplement: S1 Table — Percentages of NK subpopulations are presented as median and interquartile range (Q1-Q3). The percentage of “Total CD56+” was calculated with respect to CD3neg cells. The p values were obtained by the Mann–Whitney U test. Legend: a undetectable viral load. (DOC) [file pone.0121019.s002.doc]

**S1 Table**

| **Clinical Variable** | | **Total CD56^+^** | | **CD56^dim^** | | **CD56^bright^** | | **CD56^neg^ CD16^+^** | |
| --- | --- | --- | --- | --- | --- | --- | --- | --- | --- |
| Age  (years) | < 43 | 43 (33.5-56.8) | p=0.430 | 65.1 (52.8-69.6) | p=0.512 | 5.5 (3.5-8.5) | p=0.851 | 29.9 (22.4-35.4) | p=0.626 |
|  | ≥ 43 | 50.5 (38.6-63.4) |  | 69 (52.8-76.9) |  | 5.9 (3.7-7.4) |  | 26 (16.6-41.2) |  |
| AIDS in past (criteria) | AIDS | 51.2 (24.2-63) | p=0.648 | 69.9 (48-77.4) | p=0.708 | 6.1 (3.4-7.4) | p=0.722 | 23.2 (17.9-43.5) | p=0.893 |
|  | Non-AIDS | 46.5 (37.6-70.7) |  | 65.8 (52.9-74) |  | 5.5 (3.5-8.3) |  | 29.3 (22.1-36.9) |  |
| Nadir CD4 (cel/mL) | < 258 | 50.2 (37.6-63.6) | p=0.726 | 66.1 (49.8-77.5) | p=0.908 | 6.1 (4.4-7.6) | p=0.461 | 27.2 (17.7-40.9) | p=0.999 |
|  | ≥ 258 | 46.5 (37.2-60.5) |  | 66.3 (54.5-74) |  | 4.9 (3.2-8.7) |  | 29.3 (22.1-32.6) |  |
| Current CD4 (cel/mL) | < 652 | 50.6 (37.3-71.4) | p=0.144 | 66.7 (53.3-77.8) | p=0.667 | 6.4 (4.5-8.9) | p=0.055 | 25.1 (16-34) | p=0.224 |
|  | ≥ 652 | 45.3 (28.5-52.1) |  | 66.1 (52.6-70.9) |  | 4.6 (3.2-6.1) |  | 30.2 (22.2-41.5) |  |
| Increase CD4 (cel/mL) | < 458 | 50.5 (37.3-72.2) | p=0.267 | 68.4 (53.3-77) | p=0.531 | 6 (3.6-8.8) | p=0.377 | 26.6 (15.5-30.9) | p=0.158 |
|  | ≥ 458 | 46.5 (35.1-55.3) |  | 65.8 (52.6-72.5) |  | 5.8 (3.3-6.3) |  | 30.2 (21.9-42.8) |  |
| UVL^a^  (months) | < 85 | 45.3 (35.1-63.3) | p=0.855 | 67.8 (52.9-75.9) | p=0.728 | 6.2 (2.9-8.7) | p=0.583 | 27.3 (17.7-35) | p=0.478 |
|  | ≥ 85 | 48.9 (38.1-67.7) |  | 64.35 (52-73.6) |  | 5.8 (4.1-6.2) |  | 29.4 (21.9-41.3) |  |
| Healthy donors | | 70.4 (48.3-81.1) |  | 80.9 (75.6-82.9) |  | 5.1 (4.5-7.2) |  | 13.7 (10.7-16.7) |  |
